# Editorial: Nutritional guidelines for diabetic patients

**DOI:** 10.3389/fnut.2024.1415419

**Published:** 2024-05-03

**Authors:** Audesh Bhat

**Affiliations:** Center for Molecular Biology, Central University of Jammu, Samba, Jammu and Kashmir, India

**Keywords:** diabetes, nutritional guidelines, nutrient deficiencies, micronutrients, diabetes complication

Patients with diabetes not only suffer from high blood glucose levels and its associated complications, but also from nutritional deficiencies, especially of micronutrients due to dietary restrictions and excessive loss of body fluids (1, 2). Imbalanced protein levels are also of concern, as normal protein levels are equally necessary for better management of diabetes and its associated complications. On the other hand, excessive nutrition and its associated health conditions, such as overweight and obesity are known causative factors of diabetes. Thus, a proper nutritional guideline for diabetic patients is not only necessary for the management of diabetes but also to ensure that the patients build and improve their health. This Research Topic entitled “*Nutritional guidelines for diabetic patients*” was launched with the aim at providing a single platform to the leading scientists, clinicians and behavior scientists engaged in the field of diabetes and nutrition to share their findings and opinions on the subject. This editorial summarizes the contribution to this Research Topic. A total of six articles; one original research, one clinical trial, one study protocol, one systematic review, and one prospective were selected for publication among the numerous submissions received. A brief summary of each contribution is highlighted below.

In the first accepted article, Isaksson et al. presents a study protocol for a randomized cross-over study. Using this protocol, the authors aimed at evaluating the effects of moderate carbohydrate intake (30% of total energy from carbohydrates) on glucose levels in type 1 diabetic patients with inadequate glucose control, as against the 50% of total energy from carbohydrates traditional diet. The study outcomes were published independently in 2023 (3), showing a significant decrease in the mean glucose levels in the moderate carbohydrate group when compared with the traditional diet group (*p* < 0.001) with no incidence of hypoglycemia or ketosis.

Volek et al. presents a prospective in the second article of the Research Topic on nutrition security, highlighting the importance of having access to food that promotes overall wellbeing, including management of diabetes. The authors have argued in support of a low-carbohydrate diet based on the available evidence and inclusion of such guidelines in the Dietary Guidelines for Americans. As per the available evidence, adhering to such dietary pattern improves blood glucose and insulin control, maintains good lipid profile body weight, and lowers blood pressure and inflammatory markers.

Healthy and balanced eating along with a routine physical activity are considered as vital factors in the management of diabetes and its associated complication, including cardiovascular diseases (CVDs) (4). Zeinalabedini et al. in their cross-sectional study on 460 type 2 diabetic (T2D) patients following healthy eating index-2015 (HE1-2015) (5) reported a 50% reduction in the body roundness index (BRI) and atherogenic index of plasma (AIP) risk. This reduction was also accompanied by a reduced Castelli risk index-1 and 2, albeit with a marginal significance. This cross-sectional study further highlights the significance of adhering to a healthy diet regimen to reduce the risk of T2D and its complications.

Several decades of research have led to the identification of various plant based secondary metabolites with a potential to treat diabetes (6, 7). To assess the impact of rutin (3,3′,4′,5,7-pentahydroxyflavone-3-rhamnoglucoside), a natural flavonoid glycoside on blood pressure, antioxidant levels and several other parameters in T2D patients, Bazyar et al. conducted a clinical trial on 50 patients; 25 received rutin and 25 placebo. The authors observed a significant improvement in blood pressure and antioxidant levels in T2D patients after 3 months of 1g/day (500mg rutin+ 500 mg other ingredients) consumption. This data further adds to the plethora of data, highlighting the significance of plant based secondary metabolites in the treatment of T2D and associated complications.

The 5th article authored by Sun et al. represents a systematic review of clinical trials on the use of soy protein to mitigate diabetic nephropathy. Data from a total of 6 shortlisted randomized clinical trial was analyzed and revealed a statistically significant improvement in the kidney function parameters, such as serum creatinine, glomerular filtration rate, blood urea nitrogen, and total urine protein in both 35% and 100% soy protein categories when compared with the 0% soy protein group. The significance of certain nutrition(s) in mitigating the side effects of hyperglycemia, including nephropathy is further strengthened by this review, which clearly shows a beneficial effect of including soy protein in the diet of diabetic patients.

In the final article, Prinz presents a mini review on sweetness preference and its impact on energy impact and body weight. This interesting review summarizes the available literature on modulation of sweetness preference as a means to control body weight and obesity. The author concluded that the hypothesis lacks a strong evidence based on scientific data and highlights the need for more comprehensive research to establish any link between sweetness modulation and body weight control.

## Author contributions

AB: Conceptualization, Writing – original draft, Writing – review & editing.
